# Three-Dimensional Culture Systems in Neuroblastoma Research

**DOI:** 10.3390/organoids4020010

**Published:** 2025-05-08

**Authors:** Piotr Jung, Adam J. Wolpaw

**Affiliations:** 1 Division of Oncology, Children’s Hospital of Philadelphia, Philadelphia, PA 19104, USA; 2 Department of Pediatrics, University of Pennsylvania School of Medicine, Philadelphia, PA 19104, USA

**Keywords:** neuroblastoma, 3D culture, spheroids, tumorspheres, organoids

## Abstract

Basic and translational cancer biology research requires model systems that recapitulate the features of human tumors. While two-dimensional (2D) cell cultures have been foundational and allowed critical advances, they lack the organizational complexity, cellular interactions, and extracellular matrix present in vivo. Mouse models have thus remained the gold standard for studying cancer. In addition to high cost and low throughput, mouse models can also suffer from reduced tumor heterogeneity and species-specific differences. Three-dimensional (3D) culture models have emerged as a key intermediary between 2D cell lines and mouse models, with lower cost and greater flexibility than mouse models and a more accurate representation of the tumor microenvironment than 2D cell lines. In neuroblastoma, an aggressive childhood cancer, 3D models have been applied to study drug responses, cell motility, and tumor–matrix interactions. Recent advances include the integration of immune cells for immunotherapy studies, mesenchymal stromal cells for tumor–stroma interactions, and bioprinted systems to manipulate matrix properties. This review examines the use of 3D culture systems in neuroblastoma, highlighting their advantages and limitations while emphasizing their potential to bridge gaps between in vitro, preclinical, and clinical applications. By improving our understanding of neuroblastoma biology, 3D models hold promise for advancing therapeutic strategies and outcomes in this childhood cancer.

## 1. Introduction

Research on the mechanisms governing cellular physiology and pathology has significantly benefited from the development and application of model systems. The foundation of cell culture dates back to 1907, when Harrison conducted pioneering experiments on the origin of nerve fibers, marking the first successful cultivation of cells outside the body [1,2]. Cancer cells were famously first cultured by George Gey, who established the Hela cell line from a biopsy of the cervical cancer of Henrietta Lacks [3,4]. Cell culture techniques, now referred to as two-dimensional (2D, monolayer) cell culture, have evolved but remain indispensable tools in biology [5,6].

Two-dimensional cell culture has been particularly influential in cancer research, providing a simple and accessible platform for studying cellular behavior [7]. Widely utilized cell lines offer distinct advantages: They are readily available, cost-effective, easy to handle, and capable of producing highly reproducible data. Despite its widespread use and utility, 2D cell culture presents significant limitations. These systems fail to capture the structural complexity and microenvironment of tissues in vivo, limiting their ability to model the behavior of cells within a living organism [8]. Two-dimensional cultures rapidly lose cell–cell and cell–matrix interactions, disrupting key signaling cascades essential for in vivo cell function [9]. These signaling pathways can be important in proliferation, migration, and apoptosis, all highly relevant to tumor biology and therapy [10–12]. While 2D cancer cells can grow robustly, this can also allow them to contaminate each other, and cross-contamination of cell lines has been reported to be as high as 25% [13,14]. Prolonged passaging can also lead to progressive genetic and phenotype evolution and further deviation from the original tumor [8].

Humans have used animals to model human biology going back to ancient Greece [15]. The first animal models of cancer were used in the early 20th century and included Peyton Rous’s study of viral transmission of sarcomas in chickens and the demonstration of chemical carcinogenesis in rabbits by Katsusaburo Yamagiwa and Koichi Ichikawa [16,17]. Mouse models have come to dominate modern cancer research due to their ease of use and the ability to genetically modify them. Mouse models of cancer are widely regarded as the gold standard for mimicking human biology in cancer research, providing valuable insights into tumor behavior and therapeutic responses within a living system. Mice share several anatomical, cellular, and molecular features with humans that are essential for cancer development, and approximately 80% of mouse genes have human orthologs [18]. Cancer models in mice include transgenic tumor-prone mice, carcinogen-induced models, syngeneic transplant models, and human tumors cells grown in immunodeficient or humanized mice directly as patient-derived xenografts or indirectly as cell line-derived xenografts. Each of these systems has advantages and disadvantages, but all suffer from high cost, low throughput, and the utilization of living animals.

In recent years, three-dimensional (3D) cancer cell culture has become a key intermediary between traditional 2D cell lines and mouse models [19,20]. These models provide some of the flexibility and throughput of cell lines while more closely mimicking the in vivo microenvironment, including cellular architecture, gene expression, metabolism, and signaling pathways [21–23]. Three-dimensional cancer models have been used extensively in a variety of cancers, particularly epithelial cancers like colon, breast, liver, pancreas, prostate, ovarian, lung, and gastric cancers [10,21,23–31]. Three-dimensional cultures can be grown from human or murine tumors and formed in suspension culture [10,28] or embedded in a scaffold formed by the addition of native or artificial matrix components [23,24]. To more closely mimic tumors, 3D cultured tumor cells have been grown together with other cell types, including fibroblasts [32,33] and immune cells [28,34,35].

Three-dimensional culture has been used to study basic mechanisms of tumorigenesis [26,36], test existing therapies [37–40], screen for new therapies [25], and develop personalized treatment regimens [41], among other applications. Tumorigenesis has been studied by introducing genetic alterations into non-cancerous human tissue organoids, then examining the impact on the proliferation of the organoids and their ability to form tumors in immunodeficient mice [36,42,43]. Such studies have functionally tested the ability of mutations found in human tumors to drive tumorigenesis in human cells in a physiological cell context, something not previously possible. While not as easy to work with as 2D cell lines, 3D culture allows the throughput necessary to test drug responses. This has been used to predict patient therapy response, particularly in identifying patients likely or unlikely to respond to standard of care treatments [44–46] and to identify biomarkers of responders [41]. Three-dimensional cultures have also been used in large-scale drug testing. This includes small molecules [25], but also antibody-based therapeutics [47]. Indeed, Herpers and colleagues screened colon cancer organoids and identified a bispecific antibody targeting EGFR and LGR5 using a high-content-based platform. They went on to demonstrate its efficacy in organoids and patient-derived xenografts. This antibody (Petosemtamab) was subsequently demonstrated to have efficacy in an early-phase clinical trial [48].

This review will describe the current state of 3D culture systems in neuroblastoma, a pediatric tumor arising from the developing peripheral nervous system. We begin with a discussion of 2D cell culture and in vivo models in neuroblastoma, including patient-derived xenografts and genetically engineered models. We then describe 3D culture systems and their application in neuroblastoma research. These include the most basic models that include only tumor cells as well more complex models that include other cell types or added matrix components. We conclude with a discussion of the limitations associated with current 3D culture methodologies and how 3D culture can continue to improve our understanding and treatment of this complex disease.

## 2. Neuroblastoma 2D and In Vivo Models

### 2.1. Neuroblastoma

Neuroblastoma is the most prevalent extracranial solid tumor in children. It accounts for between 6 and 10% of all pediatric cancers and about 11% of cancer-related deaths in children [49,50]. Neuroblastomas arise from migrating neural crest cells. Primary lesions are found in the adrenal medulla or the paraspinal sympathetic ganglia of the neck, chest, abdomen, or pelvis [51]. Neuroblastoma is typically sporadic, with familial cases accounting for 1–2% of diagnoses [51]. Metastases are present in about 50% of patients at diagnosis, with bone marrow metastases as the most common location. Metastases are also found in bones and regional lymph nodes, while the involvement of the central nervous system and lungs is less common [52,53].

Neuroblastoma exhibits remarkable heterogeneity in clinical behavior, with some tumors proving fatal despite extensive, multimodal, and toxic treatments and others cured with minimal or even no therapy, including some special cases of metastatic disease [54,55]. Patients are risk-stratified according to a number of characteristics, including age, stage, pathological grade, *MYCN* amplification status, and the presence of segmental chromosomal aberrations. Low and intermediate groups have 5-year survival of greater than 85–90% while outcomes are still poor for high-risk patient with approximately 50% surviving long term [52,56]. Thus, despite significant progress in therapeutic and research efforts, the prognosis for high-risk neuroblastoma remains poor, necessitating innovative approaches to study neuroblastoma biology.

### 2.2. Neuroblastoma Cell Lines

Short-term culture of neuroblastoma cell lines dates back to the 1940s [57], even prior to the establishment of Hela cells. Murray and Stout described the morphology of eight pairs of primary and metastatic tumors in 2D culture, features which they argued could be used for diagnosis [57]. Neuroblastoma cells generally grow very well in culture and large numbers of cell lines have since been established. This has allowed researchers to define and study the differential impact of specific biologic and genetic features [58] and perform large scale experiments, including RNAi [59], CRISPR-Cas9 [60], and small molecule screens [61,62].

The large number of available cell lines has also allowed the appreciation that neuroblastoma cells can adopt different cellular morphologies in culture, sometimes even between cells in the same dish [63–65]. These cell types have been divided into three categories: neuroblastic (N-type), substrate-adherent (S-type), and intermediate (I-type), which combines characteristics of both N- and S-types [66,67]. More recently, these morphologic differences in cells aided in the transcriptional and epigenomic definition of two lineage states, an adrenergic or noradrenergic state and a mesenchymal or neural crest stem cell-like state [68,69]. These states have different super enhancer landscapes and core regulatory circuits of transcription factors [70], distinct surface proteins that may serve as immunotherapeutic targets [71], and different susceptibility to immune-cell killing [72,73]. The role of these cell line-defined states in human tumors is still debated, but there is evidence that they are relevant in patient tumors [74,75].

Despite their continued value, neuroblastoma cell lines suffer from the same drawbacks as 2D cultures in other tumor types in their failure to model the complexity of tumors [76]. This is particularly true in modeling therapy response, exemplified by PIVOT (Pediatric Preclinical In Vivo Testing Consortium) removing the in vitro component that had been part of its predecessor, the PPTC (Pediatric Preclinical Testing Consortium) [77]. Thus, there is clearly a need for a more physiological culture model.

### 2.3. Human Neuroblastoma Grown in Mice

Human neuroblastoma cell lines can be injected into immunodeficient mice as a cell line-derived xenograft (CDX) and patient tumor fragments or tumor cells can be directly implanted without culture as a patient-derived xenograft (PDX). These can be subcutaneous or orthotopic. Orthotopic injection is challenging in neuroblastoma, as the orthotopic location is the adrenal gland or the sympathetic chain, both small and not amenable to injection. However, studies have shown that implantation of tumors cells in the adrenal fat pad or under the renal capsule can produce tumors with a higher rate of metastases than subcutaneous, suggesting some improved utility to such models [78–81]. In either location, PDXs can be repeatedly passed to recipients of the following generation, and these models maintain their molecular characteristics following serial passaging [79,82,83]. Large numbers of PDXs have been developed. Many of these have been extensively characterized through efforts such as the PPTC/PIVOT [84] and the Childhood Solid Tumor Network (CSTN) [85]. Neuroblastoma PDXs are available to the research community through the CSTN and the Alex’s Lemonade Stand Foundation-funded Childhood Cancer Repository [86].

PDXs are generally considered a more accurate representation of human disease than CDXs, as the cells in a PDX have not adapted to and changed in 2D culture [82]. However, drawbacks remain. PDXs are grown in immunodeficient mice, and functional T cells are absent from athymic nude mice, functional T and B cells are absent from NOD-scid mice, and functional T, B, and NK cells are absent from the NOD-scid-gamma (NSG) strain. This limits studies of immunotherapy. Furthermore, while the tumor microenvironment (TME) of PDXs may include cell types that resemble patient neuroblastomas, the majority of these cells are derived from mice [83]. It is unclear how human tumor cells and murine stroma can signal to each other, given the species incompatibility between many secreted signaling molecules [87]. Additionally, PDXs require mice and are thus expensive, and they are not amenable to genetic manipulation. To compensate for some of these limitations, alternative models have been developed. To allow tumor-immune interactions, immunocompromised mice have been transplanted with human hematopoietic stem cells, including in mice with humanization of cytokines to promote innate immune cell development [88]. To allow genetic manipulation, human neural crest stem cells have been isolated and manipulated to form neuroblastoma after injection into immunocompromised mice [89] or immune-competent mouse embryos [90]. These newer techniques are promising but continue to be resource intensive.

### 2.4. Murine Neuroblastoma Grown in Mice

Fully murine tumors grown in mice allow for native interactions between tumor and host and a complete and functional immune system. Such models include Genetically Engineered Mouse Models (GEMMs), carcinogen-induced tumors, and murine cancer cell lines reimplanted to create syngeneic models [91]. GEMMs are particularly suited to the study of pediatric cancers that are a result of interrupted developmental programs and have less complex genomes than most adult cancers [92]. In neuroblastoma, both GEMMs and syngeneic tumors have been used extensively. The first and most widely used neuroblastoma GEMM is the *TH-MYCN* model, driven by MYCN overexpression under the tyrosine hydroxylase (TH) promoter. This produces tumors that arise along the sympathetic chain and share key histological and genetic features with human neuroblastomas [93], including expression of the immunotherapeutic target GD2 [94]. While the *TH-MYCN* model has become essential to neuroblastoma biological and preclinical investigations, additional modifications have also been made for specific purposes. These include p53 haploinsufficiency to investigate mechanisms of chemotherapy response [95,96], the addition of mutant ALK to model response and resistance to ALK inhibition [97], and the deletion of Caspase 8 to enhance bone marrow metastases [98]. Alternative models that produce neuroblastoma in mice include simian virus 40 T-antigen expression [99,100] and dopamine β-hydroxylase (DBH) promoter-driven Cre expression leading to overexpression of MYCN [101] or LIN28B [102]. GEMMs are generally excellent neuroblastoma models but are resource- and labor-intensive to maintain breeding colonies, challenging to genetically manipulate, and not amenable to substantial throughput.

Neuroblastoma cell lines that have been used to form syngeneic models include those from the *TH-MYCN* model, particularly the 9464D line that is in the C57Bl/6 background. The C1300 murine cell line was developed from a spontaneous tumor and two derivatives of this line, Neuro2a and NXS2, are frequently used and form tumors in A/J mice [103]. These lines can be implanted subcutaneously or orthotopically [104]. Cell lines from the *TH-MYCN* model have important differences from the autochthonous tumors [94]. While amenable to genetic manipulation, these models lose important features of spontaneous tumors and remain resource-intensive compared to in vitro approaches.

## 3. Neuroblastoma In Vitro 3D Models

**Figure 1.**
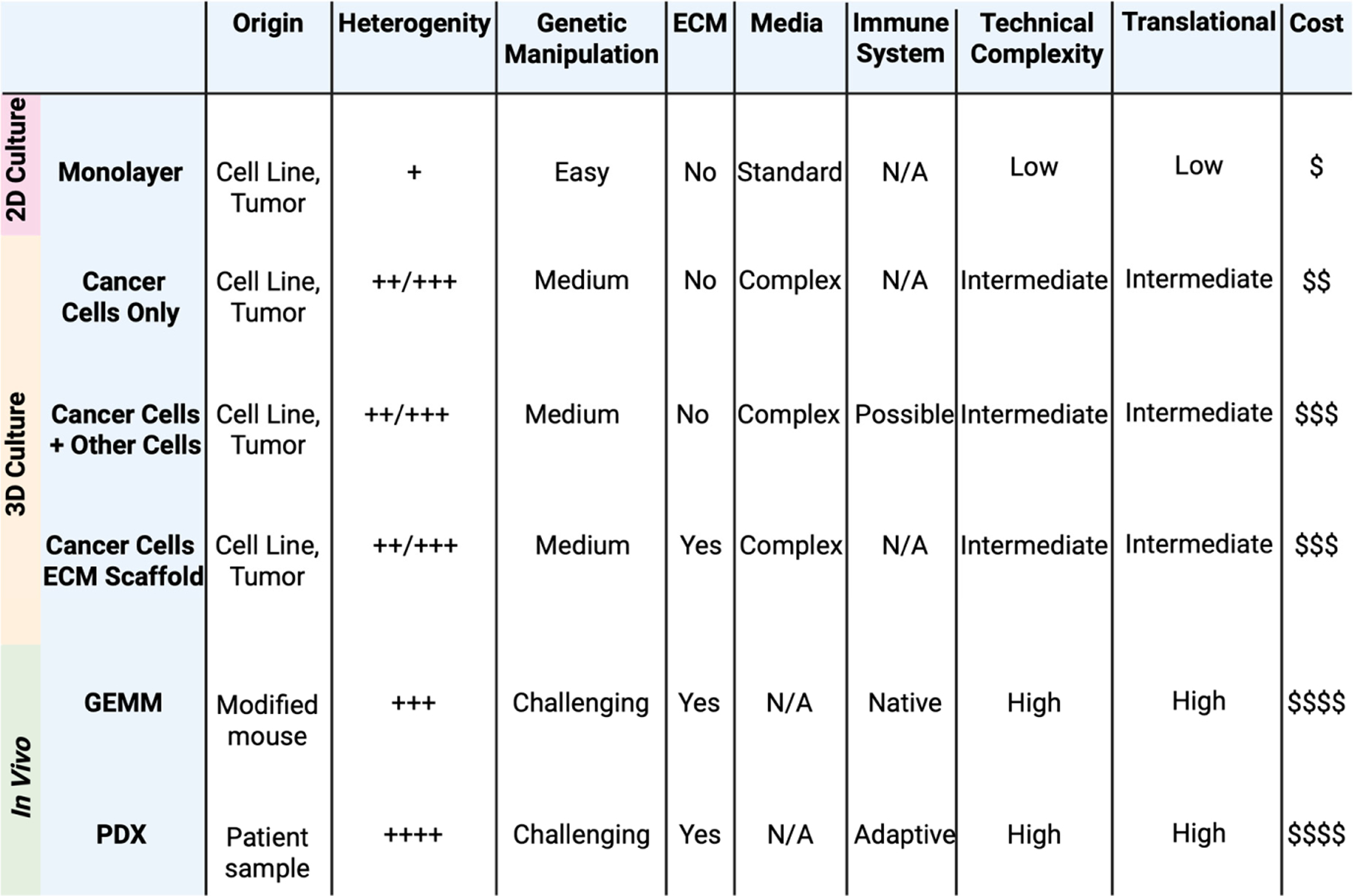
Comparison of Examples of Preclinical Models for Neuroblastoma Research. An overview of the preclinical models used to study neuroblastoma, including monolayer cultures [58–73], 3D culture systems (cancer cells alone [83,110,111,115–123,125–139], cancer cells co-cultured with other cell types such as immune cells and fibroblasts [112–114,124,140–153], and cancer cells grown in a 3D ECM scaffold [148–153,166–183]), and in vivo models (GEMMs and PDXs) [78–86,92–102]. Each model is assessed based on key attributes, including complexity, translational relevance, and ability to recapitulate the tumor microenvironment. This image was created with BioRender (https://biorender.com/ accessed on: 31 March 2025).

To address the gap between the simplistic and high-throughput monolayer culture of tumor cells and the complex but resource-intensive in vivo models, researchers have increasingly turned to 3D culture systems as a bridge, aiming to develop easy-to-reproduce, fast, and translational models to study cancer [19,31]. While 3D culture methods have existed since at least the 1970s [105], they have become increasingly utilized over the last decade. This is also the case in neuroblastoma. In neuroblastoma, some cell lines grow as multicellular spheres in suspensions even in traditional 2D culture conditions [58]. More deliberate 3D culture models have also been adopted in neuroblastoma, though they remain less characterized and established compared to those in colon or breast cancers, for example [20,106–108]. Terminology has been a challenge, in part because the term “organoid” in cancer was originally defined for epithelial cancers, and it is not straightforward to apply the same framework to non-epithelial tumors [109]. In neuroblastoma, terms such as sphere-forming cells, spheroids, neurospheres, tumor spheroids, multicellular tumor spheroids, tumorspheres, and organoids have all been used without a clear distinction between methodologies. For the purpose of this review, we will distinguish between (1) 3D cultures of neuroblastoma cells without other cell types and without an added extracellular matrix component, (2) 3D cultures of neuroblastoma cells grown with other cell types, and (3) 3D cultures of neuroblastoma cells grown with an added matrix support. We will discuss differences between the techniques that have been applied in neuroblastoma and the advantages and drawbacks they have offered in comparison to 2D culture and in vivo systems, as summarized in Figure 1.

### 3.1. Human Neuroblastoma Cells Grown Alone in 3D

The simplest 3D models involve only neuroblastoma cells without other cell types or an added extracellular matrix. While some neuroblastoma cell lines grow as unattached clusters of cells regardless of tissue culture conditions, concerted efforts have been made to force neuroblastoma cells to assume 3D structures since at least the 1980s [110]. A variety of methods have been used to achieve this. Some dissociated tumor cells will grow as spheres when grown in neural basal media (a mixture of DMEM/F12 without serum and with B27 and other growth factor supplements) [111] and some of these continuously growing 3D cell lines have been used in a number of subsequent studies [112–114]. To prevent adherence to a dish, some studies used low adherence tissue culture dishes either with neural basal media [83,115–117] or with typical serum-containing media [118–122], while another used a rotary bioreactor to create a zero-gravity condition [123]. Most studies have allowed cells to either proliferate into spheres or spontaneously aggregate, but others have centrifuged cells together [124].

3D spheres have been grown from traditional 2D cultured cell lines [118–120,123,125–129] and also directly from dissociated human tumors or PDXs without 2D culture first [83,111,115,130]. While there is an obvious appeal to avoiding 2D culture entirely and the adaptations it requires, there have not been extensive studies that compare 3D cultures that originated from cell lines to those that originated from tumors. In both cases, 3D cultured cells form spherical aggregates of cells that grow to approximately 200–700 µm in diameter. Typically, spheres of this size will develop variation in oxygenation and a necrotic core similar to a tumor [131], although this has been shown in only a minority of neuroblastoma models. Studies have shown that 3D cultured neuroblastoma cells express more cancer stem cell markers and display increased tumorigenicity compared to 2D cultures [126,128,132]. They also have transcriptomic and epigenomic features that more closely resemble tumors [83,111,115].

Neuroblastoma cells cultured alone in 3D have been used extensively to study the response to therapy and identify new therapeutic vulnerabilities. Studies have found that 3D cultures are more resistant to chemotherapy than 2D culture [128] and that 3D cultures can be used to study synergistic interactions between chemotherapy agents [133]. A recent study from the Bexell group showed that 3D cultures formed from PDXs that relapsed after chemotherapy treatment retained chemoresistance in culture compared to 3D cultures formed from chemotherapy-naïve PDXs. Furthermore, cells that survived in vitro chemotherapy treatment had similar transcriptional changes as PDXs that relapsed after in vivo chemotherapy [134]. Three-dimensional culture has also been used to study other therapeutic modalities, including antibody–drug conjugates [118], individual and combinatorial targeted therapies [120,135], differentiation therapy [129,136], and oncolytic therapy [128]. Kaess et al. used 3D culture to test the RIST treatment protocol (Rapamycin Irinotecan Sunitinib/Dasatinib Temozolomide) and showed this markedly decreased 3D cultured neuroblastoma viability [126], which is important as this regimen showed increased survival in a recent clinical trial compared to the control arm [137]. Three-dimensional cultures have also been leveraged to identify new neuroblastoma susceptibilities. Sundaramoorthy et al. identified BMX as a new therapeutic targeting by identifying transcriptional and epigenetic features common between PDXs and 3D culture but not found in 2D cell lines [115]. Three-dimensional cultures have also been adapted to high-throughput small molecule screens, which identified KSP inhibitors [138] and tyrosine kinase inhibitors with anti-neuroblastoma activity [139].

### 3.2. Human Neuroblastoma Cells Grown with Other Cell Types in 3D

While 2D co-cultures are an important tool to study cell–cell interactions, 3D models allow more native-like interactions between cell types. Tumor-immune cell interactions are the cell–cell interactions that have been most extensively studied in neuroblastoma 3D models. These models typically involve neuroblastoma sphere formation prior to the addition of immune cells. Three recent studies demonstrated the utility of 3D culture to study the activity of anti-GD2 antibodies. Kholosy and colleagues reported an assay for quantifying the death induced by an anti-GD2 antibody and peripheral blood mononuclear cells (PBMCs) [140], Troschke-Meurer et al. tested how chemotherapy alters the impact of anti-GD2 therapy and PBMCs [124], and Nguyen et al. showed that IL-15 can enhance the anti-neuroblastoma activity of anti-GD2 therapy and NK cells [141]. Other groups have studied antibody-independent killing of tumor cells. This includes NK cell killing, particularly the ability of MDM2 inhibition to enhance NK cell-mediated killing [142]. It also includes T cell killing. One study found that HDAC inhibition with etinostat increased MHC-I expression and sensitized it to killing by PRAME-specific T cells [113]. Another engineered αβ-T cells to express the γδ-T cell receptor and founds these could kill 3D neuroblastoma cultures independent of MHC-I expression [112].

Fewer studies have grown neuroblastoma cells in 3D with non-immune cells from the tumor microenvironment. One such important population in neuroblastoma is cancer-associated fibroblasts (CAFs) [143]. The Larsson group has established and used a 3D model by combining human neuroblastoma cell lines with a human dermal fibroblast cell line in low attachment plates and allowing them to form mixed spheres [144,145]. They showed that the combined spheres had a tumor-like hypoxic core and were more compact than mono-neuroblastoma spheres, and that the fibroblasts started expressing CAF markers. They then showed that inhibition of prostaglandin synthesis in fibroblasts resulted in sensitization of spheres to chemotherapy [145]. In a subsequent study, they used this model to screen for synergistic combinations with prostaglandin synthesis inhibitors. Another group made co-culture spheres in a similar fashion but with centrifugation of the cells and investigated how adrenergic and mesenchymal neuroblastoma cells interacted differently with fibroblasts [146]. This model has been more recently used by another group to test the efficacy of a novel combination therapy [147].

Another important cell type in the microenvironment of tumors is endothelial cells. Several studies have combined 3D neuroblastoma cells with endothelial cells [148–153]. In all cases, these required the addition of matrix components and/or bioprinting of scaffolds (see more in subsequent section on these techniques). Ning and colleagues used a gelatin derivative to bioprint chambers with a central cavity and microchannels, lined them with human umbilical vein endothelial cells (HUVECs), and then added neuroblastoma spheres to the cavity. The HUVECs increased the invasiveness of the neuroblastoma cells [151]. Nothdurfter and colleagues also used bioprinting with HUVEC-lined channels using a gelatin and fibrin mixture and similarly found that some neuroblastoma cells could invade the endothelial cells [152].

### 3.3. Murine Neuroblastoma Cells Grown in 3D

While the described systems using human cells have some distinct advantages, it is challenging to translate tumor-immune interactions probed in 3D culture directly into the in vivo setting. This is because such experiments would either require either immunodeficient mice or transition to murine neuroblastoma cells. For these reasons, growing murine tumor cells in 3D is appealing. Murine neuroblastoma cell lines have been used for this purpose. Neuro2A cells grow in 3D spheres in NeuroCult media and are more resistant to chemotherapy and radiation therapy compared to adherent cells [63,154]. The Fruci lab has grown 3D cultures directly from 9464D and 975A2 cells as well as from tumors formed from these lines in standard serum-containing media. By combining them with chemotherapy, they were able to demonstrate the ability of chemotherapy to sensitize to killing by splenocytes, and validate this effect in vivo [155,156].

Using spontaneous murine tumors directly in 3D culture has been more challenging than with human tumors or with human or murine cell lines. Three different groups have successfully cultured dissociated TH-MYCN tumor cells in 3D [157–161]. Two of these reported failure of non-adherent cells to grow in the type of neural basal media that had been successful with human tumors [157,159]. One was subsequently successful using a neural crest media with 12% fetal bovine serum [157] and the other by adding 15% fetal bovine serum and β-mercaptoethanol [159]. These groups went on to use these systems to investigate metabolic reprogramming in neuroblastoma [157] and the roles of PRMT1 [158] and PES1 [160] in neuroblastoma cell survival. Embaie et al. recently took a different approach and leveraged analysis of scRNAseq data from TH-MYCN tumors to design a complex culture media without serum but with a number of different growth factors and inhibitors of p38 MAPK and the TGFβ receptor. They found this media formulation promoted the growth of dissociated tumor cells in 3D when grown in Matrigel and went on to perform scRNAseq on these cells. They found that 3D culture maintained the adrenergic cell states found in the TH-MYCN tumor [161]. This last point is important, as this state is lost in TH-MYCN cell lines grown in 2D culture [94].

### 3.4. Neuroblastoma Cells Grown with an Added Matrix

A major limitation of 2D culture is the absence of a surrounding extracellular matrix (ECM), a complex network of bioactive molecules that provides structural support and signaling cues essential for cellular function, tissue organization, and tumor progression, including initiation, growth, metastases, and treatment resistance [162]. Three-dimensional culture provides an opportunity to include extracellular matrix components to more closely mimic in vivo tumors, and this has been performed extensively in other tumor types [163]. Indeed, unlike in neuroblastoma, the most commonly used 3D culture conditions incorporate the use of ECM hydrogels such as Matrigel [164], though less biologically complex scaffolds are also common, including collagen and fibrinogen [165]. In general, approaches can be divided into three groups: native TME components (such as collagen, fibrin, and Matrigel), artificial components (non-native to the TME, including cellulose and synthetic polymers), and bioengineering approaches (which can combine both native and artificial scaffolds with methods like bioprinting and microfluidic devices) [162]. While not used as extensively as in other tumor types, all three approaches have been studied in neuroblastoma.

Several research groups have incorporated native TME components in 3D neuroblastoma culture. Collagen, a molecule abundantly present in the tumor ECM, has been used to study neuroblastoma invasion [166] and collagen enriched with glycosaminoglycans and nanohydroxyapatite has been used to accurately model neuroblastoma chemoresistance [167,168]. Gelatin methacrylate (GelMA), a biomaterial derived from gelatin, can be printed and used to support 3D culture. One group used this to incorporate endothelial cells as described above [151] and another added a synthetic polymer to increase its porosity and used this system to study metastatic behavior [169]. Matrigel, derived from the secretion of Engelbreth-Holm-Swarm mouse sarcoma cells [170], is widely used in adult 3D cancer models. It has been incorporated into 3D neuroblastoma systems to promote sphere formation in both human [171] and murine 3D culture and is helpful in culturing neuroblastoma cells with endothelial cells [148].

Artificial scaffolds include those made from cellulose, a naturally occurring but non-TME component. Two independent studies have explored its applications. One study enhanced a bacterial nanocellulose scaffold with trimethyl ammonium beta-hydroxypropyl and collagen [172]. The other study chemically oxidized cellulose and modified its blend, discovering that the resulting composites enhanced cytocompatibility and promoted neurite development [173]. Other artificial scaffolds include synthetic polymers, such as polyacrylonitrile (PAN) and polyethylene glycol (PEG). PAN has been shown to enhance the survival of neuroblastoma cells [174] and PEG, when enriched with vitronectin, improved neuroblastoma cell adhesion [175]. Other studies have used scaffolds composed of silk to create hypoxic gradients [176], to study the response to integrin inhibition after incorporation of vitronectin [177], and to model the interactions between neuroblastoma cells, macrophages, and NK cells [178]. Lastly, another group used a freeze-drying technique to combine several TME components with synthetic polymers to create a porous microstructure that could support neuroblastoma cell growth [179].

More complex systems can include combinations of natural and artificial scaffolds and bioengineering methods to provide variable structure, flow, and concentration gradients. In neuroblastoma, two studies used microfluidic devices [149,150]. Liu and colleagues incorporated a decellularized ECM and microvascular endothelial cells with a microfluidic device, producing a system that could produce drug concentration gradients [149]. Villasante et al. used a microfluidic device with a collagen/fibrin scaffold and also incorporated endothelial cells [150]. Several studies have used 3D bioprinting [151,152,180,181]. In addition to the previously described studies using gelatin to incorporate endothelial cells [151–153], another group used ink made from cellulose nanofibrils, alginate, and single-walled carbon nanotubes to bioprint scaffolds for neuroblastoma cell growth [180]. Quinn et al. printed using tumor cells mixed with gelatin and sodium alginate and showed histology similar to tumors and more resistance to hypoxia and chemotherapy than 2D cell lines. Another group has published two studies [182,183] using β-tricalcium phosphate to bioprint scaffolds to model bone marrow metastases, including the incorporation of mesenchymal stromal cells. While intriguing, these systems increase cost and complexity and reduce the accessibility of these approaches for the broader neuroblastoma community while also limiting experimental design. The utility of these systems to understand neuroblastoma biology remains to be seen.

## 4. Limitations

Survival for patients with high-risk neuroblastoma has improved, but therapy is highly toxic and nearly half of patients do not survive [184]. Further advances have been hindered by in vitro models that do not reflect the tumor’s heterogeneity and its interactions with the microenvironment and in vivo models that are inflexible and difficult to use in substantial throughput. As a result, preclinical studies too often fail to predict clinical outcomes, hindering the development of effective therapies. Significant progress has been made in developing 3D models that mimic native tumors, enhancing the physiological relevance of cell-based biological and therapeutic investigations [185,186]. As we have discussed, these types of models are being adopted in neuroblastoma. It remains to be seen if these will be able to capture the heterogeneity of human tumors sufficiently to improve the success of drug development.

Creating 3D cultures for cancer, in general, presents several challenges. Serum-free media is often supplemented with more expensive alternatives [187]. Similarly, while extracellular scaffolds may improve model accuracy, they also increase the cost and complexity of the technique. Matrigel, the most commonly used matrix for 3D culture, includes growth factors in unpredictable and uncontrollable amounts, potentially influencing research results in both beneficial and detrimental ways [188]. Using 3D culture is generally more time-consuming than 2D models, often requiring increased monitoring and manipulation [189]. Due to the diverse range of 3D culture techniques, even within the same tissue or cancer type, achieving consistency remains a challenge. Analysis of cells is more difficult as well, as 3D cultures can require proteolytic degradation to separate the cells. In some cases, 3D methods have lower efficiency, shorter lifespans, and reduced replicability when compared to 2D systems [190]. Using cancer cell lines to create a 3D culture is convenient and can help in reproducibility, but this can reduce the fidelity of the model if culture in 3D does not restore the original cancer phenotype due to adaptations from prolonged 2D culture. Using freshly dissociated tumor tissue decreases accessibility and throughput, and extensive expansion may also result in further deviation from the features of the original tumor. Additionally, intra- and inter-technique variability in the size of 3D structures can impact the consistency and uniformity of the models [6,191,192]. Lastly, current models suitable for high-throughput screening are largely limited to cancer cells and a single additional cell type, such as fibroblasts [193], which may miss important cell–cell-drug interactions.

Despite these challenges, several groups have retrospectively demonstrated that drug sensitivity profiling in patient-derived 3D cultures correlates with patient response in adult cancers [41,44,46], prompting the exploration of the use of such an approach for the choice of therapy in patients [45,194,195]. These studies demonstrate the feasibility of using 3D culture to drive clinical decision making. However, additional challenges remain, including incomplete success in generating 3D cultures, prolonged time necessary to generate response profiles, and limitations in the number of drugs that can be tested [45,194,196]. Such challenges will need to be overcome to allow such testing to be adopted more universally.

**Figure 2.**
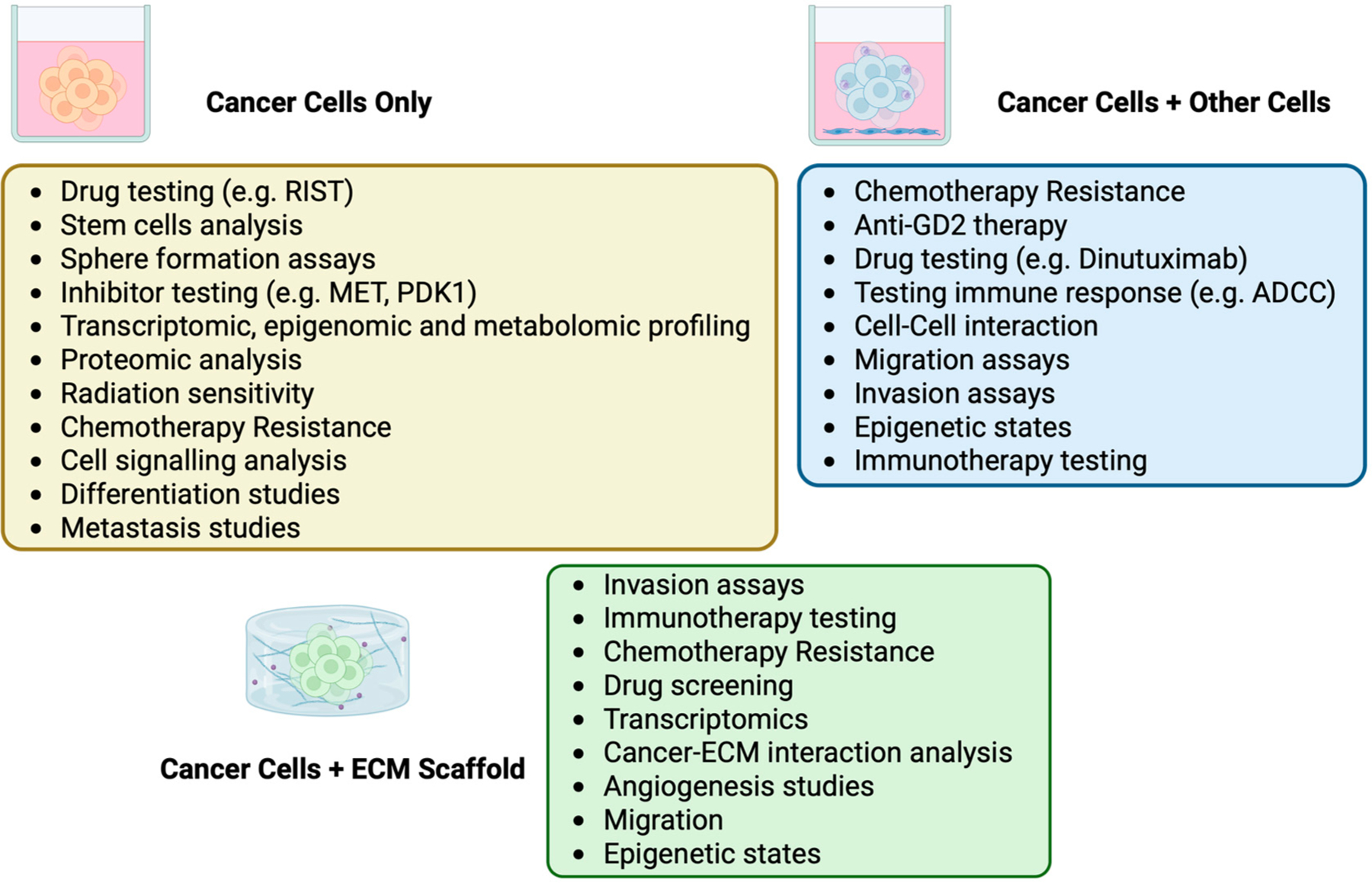
Applications of 3D Models in Neuroblastoma Research. Key applications of various 3D culture models in neuroblastoma research, including their use in drug screening, studying chemotherapy resistance, or transcriptomic and epigenomic profiling. This image was created with BioRender (https://biorender.com/ accessed on 31 March 2025).

While not as advanced as in some epithelial cancers, significant progress has been made in culturing neuroblastoma cells in 3D. These studies have performed drug screens and tested combination therapies, analyzed ECM tumor interactions, probed tumor-immune and tumor-endothelial cell interactions, and performed transcriptomic and proteomic analyses, as discussed in this review. Figure 2 summarizes some of these diverse applications. Despite these successes, many of the same challenges found with other tumors are also present for neuroblastoma. These include increased costs of media and ECM components, more time-intensive labor needs, and variability across models, conditions, and sphere sizes. Direct comparisons between the fidelity of cell line-derived and direct tumor-derived models are lacking and would be helpful to determine if less accessible tumor-derived models are necessary. There are also some more specific challenges in neuroblastoma, particularly the difficulty in growing primary murine tumors in 3D. Successful techniques have now been developed for murine tumors and should be more widely adapted. The development of direct clinical applications of patient-derived 3D culture is further behind the more common adult cancer types, in part due to the challenges inherent to the study of rare tumor types. Efforts have thus largely focused on using organoids to link genomic or transcriptomic features to drug response, rather than direct testing of patient-derived samples [130]. While direct drug-response testing may not be feasible unless histology-agnostic protocols are developed, ongoing research on 3D culture in neuroblastoma still holds great potential for advancing our understanding and treatment of neuroblastoma.

## 5. Conclusions and Future Directions

In this review, we characterize and organize the methods that have been used in 3D culture in neuroblastoma research. We identified and defined three major groups and methodologies for culturing, maintaining, and utilizing neuroblastoma 3D culture: (1) 3D culture of neuroblastoma cells without other cell types or an extracellular matrix, (2) 3D culture of neuroblastoma cells grown with other cell types, and (3) 3D culture of neuroblastoma cells grown with matrix support. We also distinguish whether these models were applied in murine or human models. We chose to use this classification to highlight the similarities and distinctions among these culture methods and avoided the words typically used (spheroids/tumorspheres/organoids), because in neuroblastoma they have been used to describe highly similar techniques without clear differences. While such terms are useful and convenient, we hope that this methodology-driven classification provides a more useful comparison of these models and their applications.

As in other cancer types, 3D culture systems in neuroblastoma have been used to make important advances in a tractable system that closely mimics in vivo conditions. These neuroblastoma models have been applied to investigate tumor heterogeneity, tumor– host cell interaction (immune cells, CAFs, and endothelial cells), metastasis, and tumor– ECM interactions. Despite these successes, there remains significant potential for further research and refinement. Future studies could focus more on integrating patient-derived immune cells to better understand immunotherapy responses. They should also aim to integrate a wider array of cell types and microenvironmental components, including diverse combinations of immune and vascular cells, to more faithfully replicate the in vivo tumor landscape. At the same time, efficiency, simplicity, and miniaturization are also important in developing better high-throughput platforms for drug screening and enabling personalized medicine approaches. Lastly, expanding the use of mouse-derived 3D models and advancing genetic engineering techniques will allow researchers to better understand anti-tumor immunity and investigate specific genetic and molecular mechanisms important in neuroblastoma progression and resistance.

As the field progresses, we expect that 3D culture technologies will become even more integral to neuroblastoma research. We expect that technological advances will enable discoveries that could lead to more effective therapies and improved outcomes for patients.

## Data Availability

No new data were created.

## Abbreviations

2D: Two-Dimensional
3D: Three-Dimensional
CDX: Cell line-Derived Xenograft
CSTN: Childhood Solid Tumor Network
DBH: Dopamine β-Hydroxylase
ECM: Extracellular Matrix
gelMA: Gelatin Methacryloyl
GEMM: Genetically Engineered Mouse Models
HDAC: Histone Deacetylase
HUVEC: Human Umbilical Vein Endothelial Cells
KSP: Kinesin Spindle Protein
NK: Natural Killer
NSG: NOD-Scid-Gamma
PAN: Polyacrylonitrile
PBMC: Peripheral Blood Mononuclear Cells
PDX: Patient-Derived Xenografts
PEG: Polyethylene Glycol
PIVOT: Pediatric Preclinical In Vivo Testing Consortium
PPTC: Pediatric Preclinical Testing Consortium
RIST: Rapamycin Irinotecan Sunitinib/Dasatinib Temozolomide
TH: Tyrosine Hydroxylase
